# Supplementary material for: Radar versus optical: The impact of cloud cover when mapping seasonal surface water for health applications in monsoon-affected India
Source: PLoS One. 2025 Jan 24;20(1):e0314033. doi: 10.1371/journal.pone.0314033 (PMC11760589; doi:10.1371/journal.pone.0314033)
Supplement: S1 Appendix — (DOCX) [file pone.0314033.s001.docx]

# S1 File. Bayesian Inference method and Valley Emphasis method.

***Bayesian Inference***

# The Bayesian inference approach establishes how likely a backscatter value is a suitable threshold by combing prior field measurements (Fig S1), a simple “noise” detection algorithm, a simplified valley test and simple reasoning about the likelihood of waterbody extent over land. Bayes’ theorem states that the likelihood of the backscatter threshold of waterbodies (β), given a satellite backscatter image (X), is proportional to the prior probability distribution of β (P(β)) times the probability of X given β.

P(β| X) ∝P(β) ×P(X | β) (S1)

#

# We set the prior based on typical Sentinel1-SAR VV backscatter time series from open year-round waterbodies, seasonal waterbodies, and year-round non-flooded values in Figure S1. We obtained P(β) by first finding the probability that a backscatter value is water (σ(β)), which is a logistic curve to backscatter (β) in Figure S1 vs a probability of 1 for waterbodies, 0.5 for seasonal waterbodies (cropland, bare fields and sand banks) and 0 for all other cover types. P(β) is the derivative of this:

σ (β)= 1/(1+e^−k×(β−β0)^)/𝜎𝛽

P(β)= σ(β)×(1− σ(β))

# P(X | β) multiples the probabilities of noise P(noise | β), total waterbody area P(area | β)×, and backscatter histogram valley magnitude P(valley | β):

P(X | β) =P(noise | β)×P(area | β)×P(valley | β)

# The noise rule is to quantify backscatter image noise, assuming waterbodies are less noisy. Similar to the manual approach, it assumes that small, single-pixel waterbodies are unlikely (i.e., it assumes waterbodies are less noisy). We calculated P(X | β)  based on the amount of “image noise” in the waterbodies suggested by β, which we defined as the ratio of the waterbody area over the perimeter of those detected waterbodies:

# P(noise| β) =(Σi,jxi,j < β )/(Σnii = 1Σnjj = 2{1 if (xi, j < β & xi,j−1 > β)  | (xi, j > β & xi,j−1 < β) } ) + {1 if (xj, i < β & xj−1,i > β)  | (xj, i > β & xj−1,i < β) }

# The area rule simply states that, given the study area is mainly land, the less waterbodies the better, and is therefore proportional the summed area great than a given threshold:

# P(area | β)∝ ∑^n^_i=1_x_i_>β

# Like valley emphasis, the valley rules assumes that areas of less density in the backscatter histograms are more likely to be our backscatter threshold and is therefore inversely proportional to the density of points around the backscatter value. We do this by summing are area with backscatter values (abitory) less than 1 away.

# P(valley | β)∝1− ∑^n^_i=1_|xi−β|<1

# We solved equation S1 using a simple accept-reject sampling technique with a normal proposal distribution (Gelman, Andrew, John B. Carlin, Hal S. Stern, David B. Dunson, Aki Vehtari 2013). The satellite image is large, so for computational efficiency, we sampled 50 chains, each chain training on a randomly selected image with dimensions 1/10th by 1/10th of the full image. We ran each chain with a “burn in” on 100 iterations to estimate the proposal distribution and 500 iterations to sample the posterior. Some selected images contained areas outside of the study area. When this occurred, we ran the chain for areas within the study area and selected a fraction of the 500 iterations proportional to the study area within the image.

# *Valley Emphasis*

# The Valley emphasis method (and Otsu method) is developed to work on histograms of integer values (e.g., 0 to 255) (Ng 2006; Li and Wang 2015; Duy 2016), while SAR-C backscatter is float values ranging, in our case from -40 dB to +5 dB. To implement valley emphasis on backscatter imagery, we first linearly scaled the -40 to +5 float values to 0 to 255 integer values and then rescaled the resulting integer threshold to float. Also, the shape of the image histogram and the resulting threshold depends on the chosen bin value, which requires manual tuning. We implemented bin sizes 0, 3, 11 and 25 (Table S1) and for further comparison, chose the thresholds most similar to our manual thresholds (i.e., those resulting from using a bin of 25).
