# Supplementary figures and images for: Radar versus optical: The impact of cloud cover when mapping seasonal surface water for health applications in monsoon-affected India
Source: PLoS One. 2025 Jan 24;20(1):e0314033. doi: 10.1371/journal.pone.0314033 (PMC11760589; doi:10.1371/journal.pone.0314033)

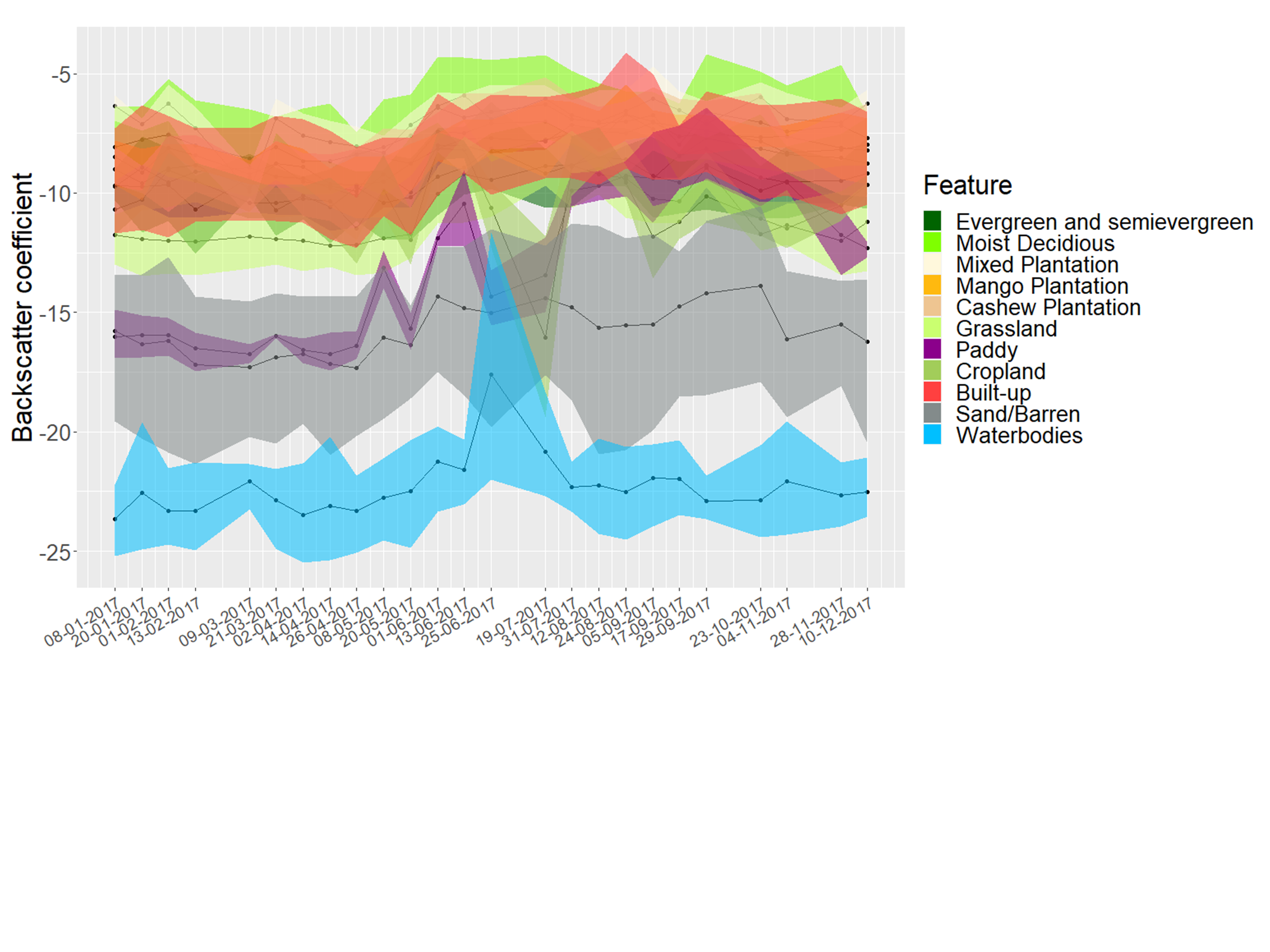

Supplement: S1 Fig — (TIF) [file pone.0314033.s002.TIF]
