# Supplementary material for: Radar versus optical: The impact of cloud cover when mapping seasonal surface water for health applications in monsoon-affected India
Source: PLoS One. 2025 Jan 24;20(1):e0314033. doi: 10.1371/journal.pone.0314033 (PMC11760589; doi:10.1371/journal.pone.0314033)
Supplement: S1 Table — (DOCX) [file pone.0314033.s003.docx]

# Table S1. Threshold values derived using the valley emphasis method for Sindhudurg Sentinel 1 SAR 2017 images, selected to represent the dry (March), monsoon (June-September) and post-monsoon (October-December) seasons.

|  | **2017/03/09** | **2017/06/25** | **2017/09/17** | **2017/10/23** | **2017/12/10** |
| --- | --- | --- | --- | --- | --- |
| **Bin 0** | -10.8317 | -8.58954 | -8.78195 | -8.981 | -9.29 |
| **Bin 3** | -14.2079 | -11.2818 | -11.6635 | -11.7959 | -12.21 |
| **Bin 11** | -16.1773 | -13.0766 | -13.5846 | -13.8066 | -14.01 |
| **Bin 25** | -17.4433 | -14.4868 | -15.6429 | -15.2811 | -16.09 |
