# Supplementary material for: Radar versus optical: The impact of cloud cover when mapping seasonal surface water for health applications in monsoon-affected India
Source: PLoS One. 2025 Jan 24;20(1):e0314033. doi: 10.1371/journal.pone.0314033 (PMC11760589; doi:10.1371/journal.pone.0314033)
Supplement: S2 Table — (DOCX) [file pone.0314033.s004.docx]

# Table S2. Waterbody mapping accuracies achieved using the manual, Bayesian inference and Valley emphasis thresholding approaches for the Sindhudurg Sentinel-1A SAR image of 10^th^ December 2017.

| **Method** | **Water**  **% UA (SE)** | **Water**  **% PA (SE)** | **Overall Accuracy**  **(%)** | **Water N^o^ ref points** |
| --- | --- | --- | --- | --- |
|  |  |  |  |  |
| Manual | 90.45 (0.67) | 97.12 (1.06) | 99.60 | 47 |
| Bayesian inference | 92.86 (0.53) | 99.89 (0.01) | 99.89 | 47 |
| Valley Emphasis | 71.69(0.89) | 99.42(0.58) | 99.59 | 47 |
