# Supplementary material for: Radar versus optical: The impact of cloud cover when mapping seasonal surface water for health applications in monsoon-affected India
Source: PLoS One. 2025 Jan 24;20(1):e0314033. doi: 10.1371/journal.pone.0314033 (PMC11760589; doi:10.1371/journal.pone.0314033)
Supplement: S3 Table — The threshold values highlighted in yellow are when largescale flooding occurred. (DOCX) [file pone.0314033.s005.docx]

**Table S3. Manual thresholds for each Sentinel-1 SAR image. The threshold values highlighted in yellow are when largescale flooding occurred.**

| **Shivamogga** | | | | **Sindhudurg** | | | | **Wayanad** | | | |
| --- | --- | --- | --- | --- | --- | --- | --- | --- | --- | --- | --- |
| **Date** | **Threshold** | **Date** | **Threshold** | **Date** | **Threshold** | **Date** | **Threshold** | **Date** | **Threshold** | **Date** | **Threshold** |
| 03-01-2017 | -17.19 | 22-01-2018 | -18.07 | 08-01-2017 | -17.76 | 03-01-2018 | -18.00 | 03-01-2017 | -17.76 | 22-01-2018 | -17.19 |
| 15-01-2017 | -17.74 | 03-02-2018 | -18.546 | 20-01-2017 | -17.23 | 27-01-2018 | -17.68 | 15-01-2017 | -17.65 | 03-02-2018 | -17.85 |
| 20-02-2017 | -17.46 | 27-02-2018 | -18.26 | 01-02-2017 | -17.17 | 15-01-2018 | -18.85 | 08-02-2017 | -17.75 | 27-02-2018 | -18.06 |
| 04-03-2017 | -17.68 | 11-03-2018 | -17.04 | 13-02-2017 | -17.45 | 08-02-2018 | -17.82 | 20-02-2017 | -18.44 | 11-03-2018 | -17.06 |
| 08-03-2017 | -17.08 | 23-03-2018 | -16.00 | 09-03-2017 | -17.61 | 04-03-2018 | -19.24 | 04-03-2017 | -18.18 | 23-03-2018 | -17.52 |
| 09-04-2017 | -17.23 | 04-04-2018 | -16.01 | 02-04-2017 | -17.97 | 09-04-2018 | -17.8 | 23-03-2017 | -17.24 | 04-04-2018 | -18.12 |
| 21-04-2017 | -18.43 | 16-04-2018 | -16.56 | 14-04-2017 | -18.99 | 03-05-2018 | -17.74 | 09-04-2017 | -17.96 | 16-04-2018 | -18.86 |
| 03-05-2017 | -17.88 | 28-04-2018 | -16.67 | 26-04-2017 | -17.57 | 27-05-2018 | -17.85 | 21-04-2017 | -18.82 | 10-05-2018 | -17.37 |
| 15-05-2017 | -17.05 | 10-05-2018 | -16.86 | 08-05-2017 | -18.19 | 08-06-2018 | -17.11 | 03-05-2017 | -18.76 | 22-05-2018 | -17.10 |
| 27-05-2017 | -17.20 | 22-05-2018 | -15.28 | 20-05-2017 | -17.6 | 20-06-2018 | -18.45 | 15-05-2017 | -17.21 | 03-06-2018 | -16.31 |
| 08-06-2017 | -17.00 | 03-06-2018 | -17.48 | 01-06-2017 | -17.14 | 02-07-2018 | -15.54 | 08-06-2017 | -17.71 | 15-06-2018 | -14.34 |
| 02-07-2017 | -13.67 | 15-06-2018 | -16.49 | 25-06-2017 | -17.33 | 14-07-2018 | -17.47 | 02-07-2017 | -15.54 | 09-07-2018 | -13.36 |
| 14-07-2017 | -15.00 | 27-06-2018 | -16.49 | 19-07-2017 | -17.1 | 26-07-2018 | -17.00 | 14-07-2017 | -18.35 | 21-07-2018 | -17.57 |
| 26-07-2017 | -17.28 | 09-07-2018 | -13.48 | 31-07-2017 | -17.36 | 27-08-2018 | -17.00 | 07-08-2017 | -18.24 | 02-08-2018 | -15.45 |
| 07-08-2017 | -17.58 | 21-07-2018 | -18.08 | 12-08-2017 | -17.64 |  |  | 14-08-2017 | -17.62 | 14-08-2018 | -13.82 |
| 19-08-2017 | -17.65 | 02-08-2018 | -15.31 | 24-08-2017 | -16.29 |  |  | 31-08-2017 | -17.96 | 07-09-2018 | -17.64 |
| 31-08-2017 | -17.83 | 14-08-2018 | -14.45 | 05-09-2017 | -15.27 |  |  | 12-09-2017 | -17.67 | 19-09-2018 | -18.76 |
| 21-09-2017 | -17.01 | 26-08-2018 | -15.29 | 17-09-2017 | -15.49 |  |  | 06-10-2017 | -18.09 | 01-10-2018 | -17.13 |
| 06-10-2017 | -17.68 | 07-09-2018 | -16.28 | 29-09-2017 | -15.65 |  |  | 17-12-2017 | -18.15 | 13-10-2018 | -18.60 |
| 17-12-2017 | -17.83 | 19-09-2018 | -16.64 | 23-10-2017 | -17.58 |  |  |  |  | 30-11-2018 | -18.69 |
|  |  | 01-10-2018 | -16.42 | 04-11-2017 | -16.37 |  |  |  |  | 24-12-2018 | -17.93 |
|  |  | 13-10-2018 | -16.78 | 28-11-2017 | -16.71 |  |  |  |  |  |  |
|  |  | 30-11-2018 | -16.12 | 10-12-2017 | -16.33 |  |  |  |  |  |  |
|  |  | 24-12-2018 | -17.65 |  |  |  |  |  |  |  |  |
| **Mean** | **-17.49** | **Mean** | **-16.97** | **Mean** | **-17.37** | **Mean** | **-17.85** | **Mean** | **-17.97** | **Mean** | **-17.75** |
| **Stdev** | **0.39** | **Stdev** | **0.80** | **Std** | **0.65** | **Stdev** | **0.68** | **Stdev** | **0.72** | **Stdev** | **1.21** |
