# Supplementary material for: Radar versus optical: The impact of cloud cover when mapping seasonal surface water for health applications in monsoon-affected India
Source: PLoS One. 2025 Jan 24;20(1):e0314033. doi: 10.1371/journal.pone.0314033 (PMC11760589; doi:10.1371/journal.pone.0314033)
Supplement: S4 Table — Total cloud cover area as estimated from JRC. (DOCX) [file pone.0314033.s006.docx]

# Table S4. Total waterbody area per district as estimated from S1A and JRC. Total cloud cover area as estimated from JRC.

| **Area of water bodies (S1A and JRC) and cloud cover(JRC) in sq.km** | | | | | | | |
| --- | --- | --- | --- | --- | --- | --- | --- |
| **Shivamogga 2017** | | | | **Shivamogga 2018** | | | |
| **Month** | **S1A** | **JRC** | **Cloud** | **Month** | **S1A** | **JRC** | **Cloud** |
| **Jan** | 299.86 | 249.03 | 1.98 | Jan | 283.77 | 274.10 | 0.12 |
| **Feb** | 263.53 | 237.05 | 0.00 | Feb | 279.57 | 242.35 | 92.72 |
| **Mar** | 277.24 | 189.42 | 0.02 | Mar | 261.80 | 217.19 | 0.00 |
| **Apr** | 239.81 | 135.74 | 0.00 | Apr | 266.50 | 177.04 | 0.01 |
| **May** | 194.92 | 77.44 | 266.67 | May | 161.14 | 18.13 | 1368.34 |
| **Jun** | 118.49 | 12.89 | 6947.28 | Jun | 238.32 | 19.13 | 7708.57 |
| **Jul** | 650.69 | 14.52 | 8420.98 | Jul | 440.65 | 11.78 | 8426.87 |
| **Aug** | 413.23 | 7.37 | 7861.01 | Aug | 702.71 | 41.30 | 7767.42 |
| **Sep** | 380.83 | 137.68 | 3708.32 | Sep | 443.12 | 343.49 | 228.86 |
| **Oct** | 370.31 | 220.10 | 2100.25 | Oct | 433.73 | 369.13 | 5.64 |
| **Nov** | NA | 279.75 | 12.78 | Nov | 418.09 | 299.48 | 371.61 |
| **Dec** | 366.31 | 287.19 | 3.72 | Dec | 362.56 | 340.83 | 0.98 |
| **Sindhudurg 2017** | | | | **Sindhudurg 2018** | | | |
| **Month** | **S1A** | **JRC** | **Cloud** | **Month** | **S1A** | **JRC** | **Cloud** |
| **Jan** | 97.00 | 62.98 | 0.00 | Jan | 124.41 | 60.12 | 0.02 |
| **Feb** | 97.98 | 61.07 | 0.00 | Feb | 78.80 | 58.86 | 0.00 |
| **Mar** | 84.66 | 57.51 | 0.00 | Mar | 75.35 | 50.61 | 102.55 |
| **Apr** | 110.15 | 45.85 | 0.01 | Apr | 81.37 | 42.85 | 0.16 |
| **May** | 94.10 | 37.30 | 124.80 | May | 198.85 | 22.46 | 69.27 |
| **Jun** | 114.30 | 3.33 | 5077.00 | Jun | 124.43 | 9.32 | 2360.39 |
| **Jul** | 102.56 | 0.10 | 5167.33 | Jul | 131.54 | 0.00 | 5170.69 |
| **Aug** | 103.59 | 9.44 | 3930.62 | Aug | 91.97 | 13.24 | 4027.03 |
| **Sep** | 119.75 | 29.71 | 1156.44 | Sep | NA | 47.53 | 621.40 |
| **Oct** | 75.00 | 33.64 | 236.45 | Oct | NA | 23.57 | 44.08 |
| **Nov** | 97.64 | 61.93 | 0.00 | Nov | NA | 59.52 | 9.32 |
| **Dec** | 72.09 | 60.74 | 0.53 | Dec | NA | 62.02 | 0.00 |
| **Wayanad 2017** | | | | **Wayanad 2018** | | | |
| **Month** | **S1A** | **JRC** | **Cloud** | **Month** | **S1A** | **JRC** | **Cloud** |
| **Jan** | 41.78 | 12.51 | 0.10 | Jan | 11.36 | 11.30 | 73.48 |
| **Feb** | 27.84 | 12.35 | 0.08 | Feb | 13.90 | 10.04 | 243.74 |
| **Mar** | 18.61 | 10.29 | 131.81 | Mar | 6.90 | 11.86 | 0.01 |
| **Apr** | 16.21 | 3.32 | 112.06 | Apr | 12.30 | 7.49 | 102.62 |
| **May** | 16.06 | 7.44 | 44.67 | May | 21.62 | 3.23 | 65.16 |
| **Jun** | 13.15 | 1.25 | 951.13 | Jun | 30.40 | 0.00 | 1992.82 |
| **Jul** | 19.70 | 0.34 | 1839.72 | Jul | 86.93 | 0.00 | 2113.57 |
| **Aug** | 20.09 | 0.00 | 2121.94 | Aug | 49.19 | 3.88 | 1568.46 |
| **Sep** | 18.28 | 11.33 | 113.61 | Sep | 22.24 | 14.18 | 13.21 |
| **Oct** | 18.48 | 13.19 | 19.12 | Oct | 17.40 | 13.58 | 8.43 |
| **Nov** | NA | 11.36 | 75.95 | Nov | 15.96 | 13.38 | 0.50 |
| **Dec** | 13.08 | 12.96 | 5.51 | Dec | 17.85 | 8.57 | 76.37 |
