# Supplementary material for: Radar versus optical: The impact of cloud cover when mapping seasonal surface water for health applications in monsoon-affected India
Source: PLoS One. 2025 Jan 24;20(1):e0314033. doi: 10.1371/journal.pone.0314033 (PMC11760589; doi:10.1371/journal.pone.0314033)
Supplement: S5 Table — (DOCX) [file pone.0314033.s007.docx]

# Table S5. Per district: Number of patches, mean patch area and patch density calculated from S1A and JRC waterbody maps.

| **Number of Patches** | | | Mean Patch Area | | | Patch Density | | |
| --- | --- | --- | --- | --- | --- | --- | --- | --- |
| **Month** | S1A | JRC | Month | S1A | JRC | Month | S1A | JRC |
| **Jan** | 31231 | 1395 | Jan | 0.91 | 17.8 | Jan | 3.7 | 0.165 |
| **Feb** | 39018 | 1389 | Feb | 0.637 | 17 | Feb | 4.62 | 0.165 |
| **Mar** | 46954 | 1127 | Mar | 0.343 | 16.8 | Mar | 5.75 | 0.134 |
| **Apr** | 65153 | 848 | Apr | 0.358 | 16 | Apr | 7.72 | 0.1 |
| **Dec** | 47282 | 1733 | Dec | 0.736 | 16.5 | Dec | 5.61 | 0.206 |
| **Number of Patches** | | | Mean Patch Area | | | Patch Density | | |
| **Month** | S1A | JRC | Month | S1A | JRC | Month | S1A | JRC |
| **Jan** | 21812 | 1812 | Jan | 1.23 | 15.1 | Jan | 2.59 | 0.215 |
| **Mar** | 40894 | 1589 | Mar | 0.622 | 13.7 | Mar | 4.85 | 0.188 |
| **Apr** | 51470 | 1311 | Apr | 0.506 | 13.5 | Apr | 6.1 | 0.155 |
| **Oct** | 10950 | 2137 | Oct | 3.74 | 17.3 | Oct | 1.3 | 0.253 |
| **Dec** | 9263 | 1749 | Dec | 3.67 | 19.5 | Dec | 1.1 | 0.207 |
| **Number of Patches** | |  | Mean Patch Area | |  | Patch Density | |  |
| **Month** | S1A | JRC | Month | S1A | JRC | Month | S1A | JRC |
| **Jan** | 21243 | 1182 | Jan | 0.415 | 4.99 | Jan | 4.11 | 0.232 |
| **Feb** | 21007 | 1175 | Feb | 0.422 | 4.87 | Feb | 4.06 | 0.23 |
| **Mar** | 19306 | 1133 | Mar | 0.392 | 4.77 | Mar | 3.73 | 0.222 |
| **Apr** | 27951 | 1066 | Apr | 0.362 | 4.06 | Apr | 5.41 | 0.209 |
| **Nov** | 19223 | 1612 | Nov | 0.46 | 3.6 | Nov | 3.72 | 0.316 |
| **Dec** | 4504 | 1602 | Dec | 1.32 | 3.54 | Dec | 0.897 | 0.314 |
| **Number of Patches** | | | Mean Patch Area | | | Patch Density | | |
| **Month** | S1A | JRC | Month | S1A | JRC | Month | S1A | JRC |
| **Jan** | 22457 | 1234 | Jan | 0.5 | 4.54 | Jan | 4.34 | 0.242 |
| **Feb** | 15345 | 1233 | Feb | 0.454 | 4.45 | Feb | 2.97 | 0.242 |
| **Apr** | 18382 | 1190 | Apr | 0.391 | 3.36 | Apr | 3.56 | 0.233 |
| **Number of Patches** | | | Mean Patch Area | | | Patch Density | | |
| **Month** | S1A | JRC | Month | S1A | JRC | Month | S1A | JRC |
| **Jan** | 11233 | 75 | Jan | 0.297 | 16.7 | Jan | 5.51 | 0.0353 |
| **Feb** | 5798 | 102 | Feb | 0.394 | 12.1 | Feb | 2.84 | 0.048 |
| **Oct** | 786 | 163 | Oct | 1.89 | 8.1 | Oct | 0.389 | 0.0766 |
| **Dec** | 1721 | 143 | Dec | 0.549 | 9.07 | Dec | 0.847 | 0.0672 |
| **Number of Patches** | | | Mean Patch Area | | | Patch Density | | |
| **Month** | S1A | JRC | Month | S1A | JRC | Month | S1A | JRC |
| **Mar** | 1334 | 111 | Mar | 0.497 | 10.7 | Mar | 0.629 | 0.0522 |
| **Sep** | 1272 | 205 | Sep | 1.42 | 6.92 | Sep | 0.628 | 0.0955 |
| **Oct** | 545 | 203 | Oct | 2.68 | 6.7 | Oct | 0.268 | 0.0955 |
| **Nov** | 784 | 193 | Nov | 1.62 | 6.94 | Nov | 0.385 | 0.0908 |
