# Supplementary material for: Radar versus optical: The impact of cloud cover when mapping seasonal surface water for health applications in monsoon-affected India
Source: PLoS One. 2025 Jan 24;20(1):e0314033. doi: 10.1371/journal.pone.0314033 (PMC11760589; doi:10.1371/journal.pone.0314033)
Supplement: Attachment — Submitted filename: response to reviewers.docx [file pone.0314033.s008.docx]

Reply to reviewers’ comments

We thank the reviewers for their valuable comments which have helped us improve our manuscript.

**Reviewer #1:**

1. It’s unclear how the Bayesian noise recognition algorithm was used to map the surface water. There should be a summary of the major contribution or improvement of the proposed algorithm.

This is a new method, and while we describe it extensively in the supplement, we agree that it deserves more description in the main paper. We have therefore improved the paragraph (lines 236 – 246):

“*The Bayesian method was developed to analyse SAR imagery for an entire district regardless of the backscatter distribution ( unimodal or bimodal). This method estimates the probability of each backscatter value being an effective threshold for detecting waterbodies. It begins with a prior distribution calculated from reference backscatter values for water and non-water (Fig S1). It then incorporates likelihoods based on image noise, total waterbody area, and backscatter histogram valley magnitude. For image noise, we assume that waterbodies are typically connected (i.e., cover more than one or two image pixels) and noisy areas in the images are less likely to be water. The total waterbody area assumes that there is generally more land than water in an image. Finally, if the threshold falls within a valley on a histogram, it's more likely to be an effective threshold. Further details about our approach, how these likelihoods are combined, and the sampling methods can be found in the supplement.”*

2. Although health issues such as water-related diseases are mentioned many times in the title and text, this article only implements water extraction. How to better correlate the extraction results with disease risks? The main scope of the paper was to quantify the impact of cloud cover on optically derived water surface maps in a monsoon region by comparing a radar derived water surface map with a popular optically derived water surface product, and to discuss the implications for disease modelling. Including the radar derived maps into disease risk modelling is not within scope. We have altered the title and text in the abstract and introduction to clarify and shift the focus.

3. Is there a method flow chart that can provide a more intuitive view of the methods used? The figures are of low resolution in the main content and are difficult to read. We have added a schematic that summarises the 3 main steps taken in our approach and explained the steps at the very start of the methods sections (lines 124-128).

Detailed comments:

Introduction
L121: (< 900m2) should be “m2” . Done

Methods
Data sources
The source of the validation data was not mentioned. We describe the collection of the validation data under ‘ accuracy assessment and since the reference data was collected by the authors there is no ‘ external source’. We have added clarified this by editing the text as follows: *“ we compared both against ‘water’ and ‘non-water’ observations collected in the field in August 2018, December 2017, and November 2018 for Shivamogga, Sindhudurg and Wayanad, respectively. The reference points were collected as part of a related, but independent land cover mapping exercise of the three districts (58).”* (lines 309- 312).

L197 “… the histogram will shift from bi-modal to unimodal …”, suggest reading following study. It proposed an adaptive thresholding approach, and the bi-model and unimodal were automatically identified to generate different thresholds. Chen, S.; Huang, W.; Chen, Y.; Feng, M. An Adaptive Thresholding Approach toward Rapid Flood Coverage Extraction from Sentinel-1 SAR Imagery. Remote Sens. 2021, 13, 4899. <https://doi.org/10.3390/rs13234899> . We have mentioned the work of Chen et al 2021 in section ‘ Available approaches’ and in our discussion. Lines

Extraction of waterbodies
L224: A new Bayesian inference approach is mentioned, which is called “a Bayesian noise recognition algorithm” in the abstract. Is there a unified name for this method? No there is not, to avoid confusion we have replaced “a Bayesian noise recognition algorithm” with the less specific term *“ a Bayesian inference approach”.* (line 233)

L227: “in section 2.5” There is no such section, please check whether the expression is correct. Done, we now refer to the “accuracy assessment section”. (Line 247)

L244: What evidence is there that manual approach is better than the Bayesian approach during the monsoon? Why not use the more accurate method? Although the Bayesian method was performing better for the post-monsoon period, without in-situ data collected in the pre-monsoon and monsoon period for the same region we have no way of evaluating its performance across seasons. However, in the monsoon season, paddy fields become a significant source of waterbody expansion. They often are small waterbodies surrounded by non-waterbodies, causing them to appear as "noise" in the Bayesian algorithm. This can bias the threshold identification, leading to an underestimation of water body number and extent. Additionally, flooded paddies have dB values that are higher than open water when the rice crop is planted and grows. To address these issues in any automated thresholding system, including the Bayesian method, adjustments need to be made to avoid biased results during the monsoon. For our comparison with the JRC product, it is important to strive for a non-biased approach (such as our manual approach), even if it may not perform as well as an automated approach.

We have clarified this in the manuscript. Lines 262-272.

L251: The units for the threshold values of backscattering coefficients are not stated in many places in the article. The unit of threshold value, dB, should be added. Done

L268: The values in the table have no units (e.g., dB, %). Done

Table 3: What does probability refer to in columns 3 and 4 in Table 3? No obvious explanation is found. The content of the table is arranged in a confusing manner. It represents the probability of the proposed threshold being effective in detecting waterbodies. We have included this explanation in the table. We have also rearranged the columns to reduce confusion.

Accuracy Assessment
L286: Why are points collected along roads when using Sentinel2-MSI as reference data? What is the distance range between the collection points and roads? Is randomly selecting points a better way?

The reference points were collected from observations in the field not from interpreting Sentinel-2 imagery. As a result, a random sampling is difficult to implement because of accessibility. We have rewritten parts of the text to make sure this is made clear. Lines 304 – 306.

L319: “km2” There are many such formatting problems. Done

**Reviewer #2:**

We thank the reviewer for their comments.

1. Title should be rewrite it’s not rich: We have changed the title to ensure it better represents the scope of the paper: “Radar versus optical: the impact of cloud when mapping seasonal surface water for health applications in monsoon-affected India.”

2. Line 32, it may better use sentinel-1 or Sentinel 1 We replaced Sentinel1 with Sentinel-1 throughout the document.

3. Introduction section is very poor. Please rewrite this within 1.5 to 2 page giving all the relevant information (Novelty, limitation, application) and recent studies. We believe our introduction (although it does not necessarily follow the structure suggested by the reviewer) clearly covers the context of the work: (1) we identify the types of surface water maps required to help understand and predict disease risk modelling; (2) we summarise past and current RS efforts to deliver this type of data; (3) we highlight the challenges with optical RS data and (4) opportunities with radar data (pointing at examples of work using optical and radar derived surface water information for disease related applications).

However, as suggested by reviewer 1, we have changed small parts of the introduction to make sure the purpose of the presented work is clearer: The main scope of the paper was to quantify the impact of cloud cover on optically derived water surface maps in a monsoon region by comparing a radar derived water surface map with a popular optically derived water surface product, and to discuss the implications for disease modelling).

4. Why the study area is selected? put your statement in study area section. We have added the following line: “To contrast between different seasonal surface water dynamics and land cover/use landscape patterns,…” Lines133-134

5. Line 188, (< ~30o) degree? It is now 30^o^

6. Figure 4b where is legend? The legend is the same for all maps shown in Figure4 and is shown in Figure 4c

7. All of the figure placed in low regulation and unclear. Make sure their better regulation and readable. We have submitted figures with high resolution.

8. Discussion should be rewrite with concise information found from the result. As suggested, we have explicitly pointed to the results where relevant and further improved our discussion.

9. Rewrite the conclusion in a informative way. We have rewritten the conclusions.

10. The title suggests a focus on health issues; therefore, there is a need for greater emphasis on how the work contributes to addressing health concerns. We have changed the title to ensure it better represents the scope of the paper: “Radar versus optical: the impact of cloud when mapping seasonal surface water for health applications in monsoon-affected India.”

11. Add some recent citation This comment is vague; we are not sure which recent citation we should include. However, we have added Chen, S.; Huang, W.; Chen, Y.; Feng, M. An Adaptive Thresholding Approach toward Rapid Flood Coverage Extraction from Sentinel-1 SAR Imagery. Remote Sens. 2021, 13, 4899. <https://doi.org/10.3390/rs13234899> as suggested by reviewer 1.

12. Enhance the quality of the writing throughout. We have checked the manuscript for long sentences and grammatical errors and edited as required (see track changes).
