# Supplementary material for: Radar versus optical: The impact of cloud cover when mapping seasonal surface water for health applications in monsoon-affected India
Source: PLoS One. 2025 Jan 24;20(1):e0314033. doi: 10.1371/journal.pone.0314033 (PMC11760589; doi:10.1371/journal.pone.0314033)
Supplement: Attachment — Submitted filename: response to reviewers - 2nd review.docx [file pone.0314033.s009.docx]

We thank the reviewers for their valuable feedback and have made further changes following this feedback.

**Reviewer #3**
***-Title: The impact of cloud -> 'the impact of clouds', or 'the impact of cloud cover'***

Title now says 'the impact of cloud cover'

***-General: Is disease spread the primary interest when looking at surface water? Are there other reasons we might be interested in this? I can imagine it's also useful for agriculture, drought monitoring, leak detection....?***

Reviewer#3 (and reviewer#5) is right to point out that mapping surface water is relevant to other applications. However, because the mapping work presented here was carried out as an integral part of a disease risk project in India, our focus was on quantifying the loss of information in an area where cloud cover is seasonal and highlighting the consequences this may have for this particular application. A comprehensive discussion acknowledging a range of applications would have required us to first establish in detail the type of surface water information required by each application (in terms of repeat frequency and spatial resolution) followed by application specific discussions covering the implications and solutions. An interesting proposition, however, not within the scope of the presented work. However, to highlight our work’s relevance to other applications, we have added the following sentence (highlighted in yellow) at the beginning of our discussion:

Line 417: “Accurate and high temporal and spatial resolution surface water mapping is vital for modelling the risks of wide-ranging water-related diseases.  It is also relevant to other applications requiring seasonal mapping, such as, monitoring water availability in water scares regions, or quantifying the contribution of seasonal and/or small lakes as carbon emitters (66) “

***-Available approach discussion (from line 211 onward):
It doesn't surprise me that bimodal histogram or otsu thresholding are unsuitable, since these methods will fail if you don't have two clear populations of pixels. It does surprise me a bit that Otsu's threshold would set its threshold at the point where it's shown in figure 4c.***

It surprised us as well. The Otsu threshold was implemented independently by two of our team members with the same result, giving us confidence figure 4c is correct.

***-Line 241: Here you state that your threshold is chosen based on whether or not there are many single pixels mapped as water. Do you think these single pixels are actually noise, or might they represent small bodies of water? Or do waterbodies below a certain size threshold not contribute to disease spread? If so, it might also be useful to use a connected components analysis and filter by size. That aside, I agree that the Bayesian approach is probably the better one.***

Radar backscatter is inherently noisy and it is standard to filter (average) across space or time to reduce the noise. Here we applied a spatial 3x3 lee filter with limited smoothing reducing some but not all of the spatial noise. So, yes the reviewer is correct to wonder why single pixels were considered as noise. After all it is important to map as small a water body as possible, as every water body is important when modelling disease spread. In practice we are compromising between dealing with a noisy data set (even after filtering) and trying to correctly map as many single pixel water bodies as possible. The Bayesian approach does not exclude single pixels, rather it assigns likelihood based on total water body size histogram valley detection, evidence from reference data collection AND noise detection. So, while small water bodies might look like noise, there are three other ways the approach can raise the likelihood of them being classified as water. In the end what we produced is a water body map that is slightly better at mapping smaller water bodies than a 30m TM based map.

We have added the following text (highlighted in yellow) - line 439 - further highlighting the importance of high(er) spatial resolution radar compared to high(er) spatial resolution optical:

“The use of higher spatial resolution optical imagery (e.g., from Sentinel-2 or Planet Labs) could resolve this, however, where disease risks are linked to the rainy season, and transmission is linked to small waterbodies, the benefits of using SAR over optical for surface water mapping are particularly pronounced,…”

***-The Sentinel approach identifies more small waterbodies. How large or small does a waterbody have to be to contribute to disease spread? If there is literature on this it may be useful to put a reference in the discussion.***

According to our social scientists conducting community household surveys in the landscape, people, livestock and wildlife, all of which are hosts for pathogens and vectors, use water-bodies as small or smaller than 10m^2^ for watering as well as larger water-body edges. So a range of water-body sizes and types will be relevant to environmental transmission, though these relationships are not often empirically described. We have included this in our discussion as follows:

Line 435: “According to our social scientists conducting community household surveys in the landscape, people, livestock and wildlife, all of which are hosts for pathogens and vectors, use waterbodies as small or smaller than 10m^2^ as well as larger water-body edges for watering. So, capturing the complete range of water-body sizes is key to understand environmental transmission.”

We have also enhanced the importance of access to high spatial resolution by

1. adding ‘high spatial resolution’ to the following sentence – line 439: “The use of higher spatial resolution optical imagery (e.g., from Sentinel-2 or Planet Labs) could resolve this, however where disease risks are linked to the rainy season, and transmission is linked to small waterbodies, the benefits of using high spatial resolution SAR over optical for surface water mapping are particularly pronounced…”
2. and including in our conclusions – line 521: “Still, without meter resolution SAR imaging, many very small water bodies, relevant for disease risk modelling and other applications, will remain unmapped.”

***-Figure 5: What do you mean here by GCP? This is not an acronym I can find elsewhere in the paper.***

Thank you for pointing this out. We have updated the figure, replacing GCP (ground control points) with GRP (ground reference points) and included the acronym in the figure title:

“ Fig 5. Distribution of ground reference points (GRF) displayed with the S1A water body map…”

***-I may have missed it in this 94-page pdf, but I can't find any figure descriptions. Unless it conflicts with the guidelines for the journal I'd like to see a brief description of each figure below the figure***.

This is to do with how the material is being submitted online. While the manuscript itself contains the figure titles, the figures are submitted without title.

**Reviewer #4:**

***I would suggest the authors to add a paragraph in the conclusion section mentioning the limitations and recommendations for future studies.***

We have added a final conclusion at the end:

Line 521: “Still, without meter resolution SAR imaging, many very small water bodies, relevant for disease risk modelling and other applications, will remain unmapped.“

***Also, the authors should more clearly explain the novelty or research gap in the abstract.***

We have edited the abstract to emphasize the lack of studies quantifying the impact of cloud cover: “A critical drawback of the latter is data loss due to cloud cover, however few studies have quantified this.”

**Reviewer #5:**

***The manuscript has improved significantly after the 1st review. However, I have reservations still on one point raised by the previous reviewer also. The title of the manuscript and the discussion of the health related issues in the manuscript. The manuscript is supposed to provide improvements on the models for the mapping of water bodies in the cloud cover time using the SAR data. The method is somewaht improvement from the previous works. But I feel that the previous works on SAR are also quite accurate in mapping the water bodies so why this new complex method? The comparision with optical data does not make much sense here as everyone know optical data will be useless during the cloud time. This if the authors want to prove that the proposed method is more acurate than the previous method they have to compare it will the existing method using the SAR data.***

Actually, our main focus of the presented paper is quantifying data loss caused by cloud cover in optical imagery. The most important point is that when an application (in our case disease risk mapping) requires seasonal mapping in regions where cloud cover is dense, the consequences of relying on optically derived maps is substantial. As far as we are aware no other published study has quantified the data loss. We felt it was important to highlight this as, although the impact of cloud cover on optical imagery may be obvious to remote sensing experts, this is not necessarily the case for non-experts, and many are using optically derived products in their applications. We have made this message stronger in the abstract and conclusions as follows:

Abstract: “A critical drawback of the latter is data loss due to cloud cover, however few studies have quantified this.”

Conclusions : “Even though the 30m JRC Global Surface Water product achieves high surface water mapping accuracies when there is no cloud, using Sentinel-1A SAR data becomes essential for areas with highly dynamic surface waters and cloudy/rainy seasons as the impact of cloud cover on optically derived seasonal information is substantial.”

Although the core of the work presented is a RS-based study, the main, initial objective was not to develop a new (better) method using SAR, but to apply an easy to implement SAR based alternative for disease risk modelling and to quantify the loss of information due to cloud. The Bayesian approach evolved to check the validity of going manual and consequently an important main point is that manual is as good as many of the other approaches developed to threshold SAR data. Also, the Bayesian approaches (including ours) require priors which are often manually generated. Finally, we do not accept our Bayesian approach as more complex than any of the others suggested. As a matter of fact, our approach (e.g., applicable to other landscapes in the future) has the potential to remove the need to tile and search for bi-modal distributions, making its implementation less complex.

***Further the application of the produced water map could be anything if authors what it to be only in the case of health related issues they will have to quantify the same i.e. what will be the quantitative difference when this map is used in standerd health model or if the other water maps are used. This it is recommended to remove this part completely from the manuscript.***

See our reply to Reviewer#3. Also the reviewer’s suggestion to quantify the impact of the data loss on the disease risk models, although an interesting follow-on, would substantially expand the work and is therefore not within the scope of this particular paper.
